# Genetic Diversity of *Mycobacterium tuberculosis* Isolates from Tibetans in Tibet, China

**DOI:** 10.1371/journal.pone.0033904

**Published:** 2012-03-30

**Authors:** Haiyan Dong, Li Shi, Xiuqin Zhao, Ba Sang, Bing Lv, Zhiguang Liu, Kanglin Wan

**Affiliations:** 1 State Key Laboratory for Infectious Disease Prevention and Control, National Institute for Communicable Disease Control and Prevention, Chinese Center for Disease Control and Prevention, Changping, Beijing, People's Republic of China; 2 Tibet Autonomous Region People's Hospital, Lhasa, Tibet Autonomous Region, People's Republic of China; St. Petersburg Pasteur Institute, Russian Federation

## Abstract

**Background:**

Tuberculosis (TB) is a serious health problem in Tibet where Tibetans are the major ethnic group. Although genotyping of *Mycobacterium tuberculosis* (*M. tuberculosis*) isolates is a valuable tool for TB control, our knowledge of population structure of *M. tuberculosis* circulating in Tibet is limited.

**Methodology/Principal Findings:**

In our study, a total of 576 *M. tuberculosis* isolates from Tibetans in Tibet, China, were analyzed via spoligotyping and 24-locus MIRU-VNTR. The Beijing genotype was the most prevalent family (90.63%, n = 522). Shared-type (ST) 1 was the most dominant genotype (88.89%, n = 512). We found that there was no association between the Beijing genotype and sex, age and treatment status. In this sample collection, 7 of the 24 MIRU-VNTR loci were highly or moderately discriminative according to their Hunter-Gaston discriminatory index. An informative set of 12 loci had similar discriminatory power with 24 loci set.

**Conclusions/Significance:**

The population structure of *M. tuberculosis* isolates in Tibetans is homogeneous and dominated by Beijing genotype. The analysis of 24-locus MIRU-VNTR data might be useful to select appropriate VNTR loci for the genotyping of *M. tuberculosis*.

## Introduction

Tuberculosis (TB) remains a major health problem in China. A 2000 national TB epidemiology survey conducted in China reported the average prevalence of TB amounts to 367 per 100,000 (0.0036%), with an estimated 4.5 million active pulmonary TB patients and 1.5 million new infections a year [1]. The prevalence rate of TB in western China was higher than central and eastern regions of the country. Furthermore, the increase of multi-drug resistant (MDR) TB in China inhibits the cure/treatment of the disease.

Tibet Autonomous Region (Tibet) is located in the Qinghai-Tibet Plateau of western China and Tibetans account for more than 90% of this population. Based on the 1990 national TB epidemiology survey in China, the prevalence rate of TB in Tibet (1203.06/100,000) was higher than anywhere else in China. In 2005, 4291 active pulmonary TB cases were reported in Tibet, posing a serious threat to the public health in Tibet [2].

Molecular typing of *M. tuberculosis* strains has proven to be a valuable tool for TB control in terms of tracking transmission chain, detecting suspected outbreaks, and identifying successful clones [3]. During the last few years, several PCR-based methods have been developed including spoligotyping and mycobacterial interspersed repetitive unit-variable number tandem repeat typing (MIRU-VNTR). Spoligotyping is a rapid and convenient typing method that is useful for the recognition of *M. tuberculosis* complex lineages on the basis of the presence or the absence of some specific spacer sequences in the direct repeat region of the mycobacterial genome [4], [5]. In addition, the database SITVIT2 (http://www.pasteur-guadeloupe.fr:8081/SITVITDemo) has been developed for *M. tuberculosis* complex lineage identification by utilizing spoligotype signature matching [5]. Nevertheless, spoligotyping is not informative for Beijing genotype strains because almost all strains in this genotype share an identical spoligotype [6], [7]. The MIRU-VNTR method has proven to be faster and easier to perform and has been considered a good alternative to the gold standard method IS*6110*-RFLP [8], [9], [10], [11]. The 24-locus MIRU-VNTR method has been proposed to be the reference in standard typing and several reports have shown its appropriateness for population-based studies of TB transmission [10], [12], .

Our study aimed to examine the strain diversity and the prevalence of Beijing genotype strains in Tibet. The power of the 24-locus MIRU-VNTR scheme to differentiate the Beijing genotype strains from Tibet was also investigated.

## Results

### Description of isolates

**Figure 1:**
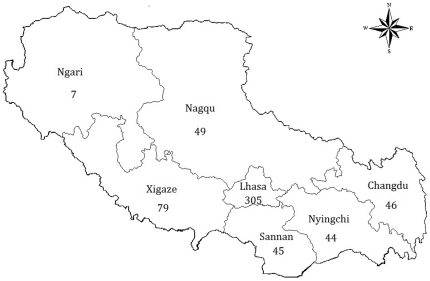
Map of Tibet showing the distribution of *M. tuberculosis* included in the present study (the number indicate the absolute number of isolates per region).

A total of 576 *M. tuberculosis* isolates from 329 male and 247 female Tibetan TB patients with a median age of 34 (range 8 to 85 years) were included in the study. These isolates comprised of 305 isolates from the Lhasa region, 79 from the Xigaze region, 49 from the Nagqu region, 46 from the Changdu region, 45 from the Sannan region, 44 from the Nyingchi region and 8 from the Ngari region (Figure 1). New cases consisted of 317 patients, and the remaining 259 patients were retreatment patients.

### Spoligotyping

**Figure 2:**
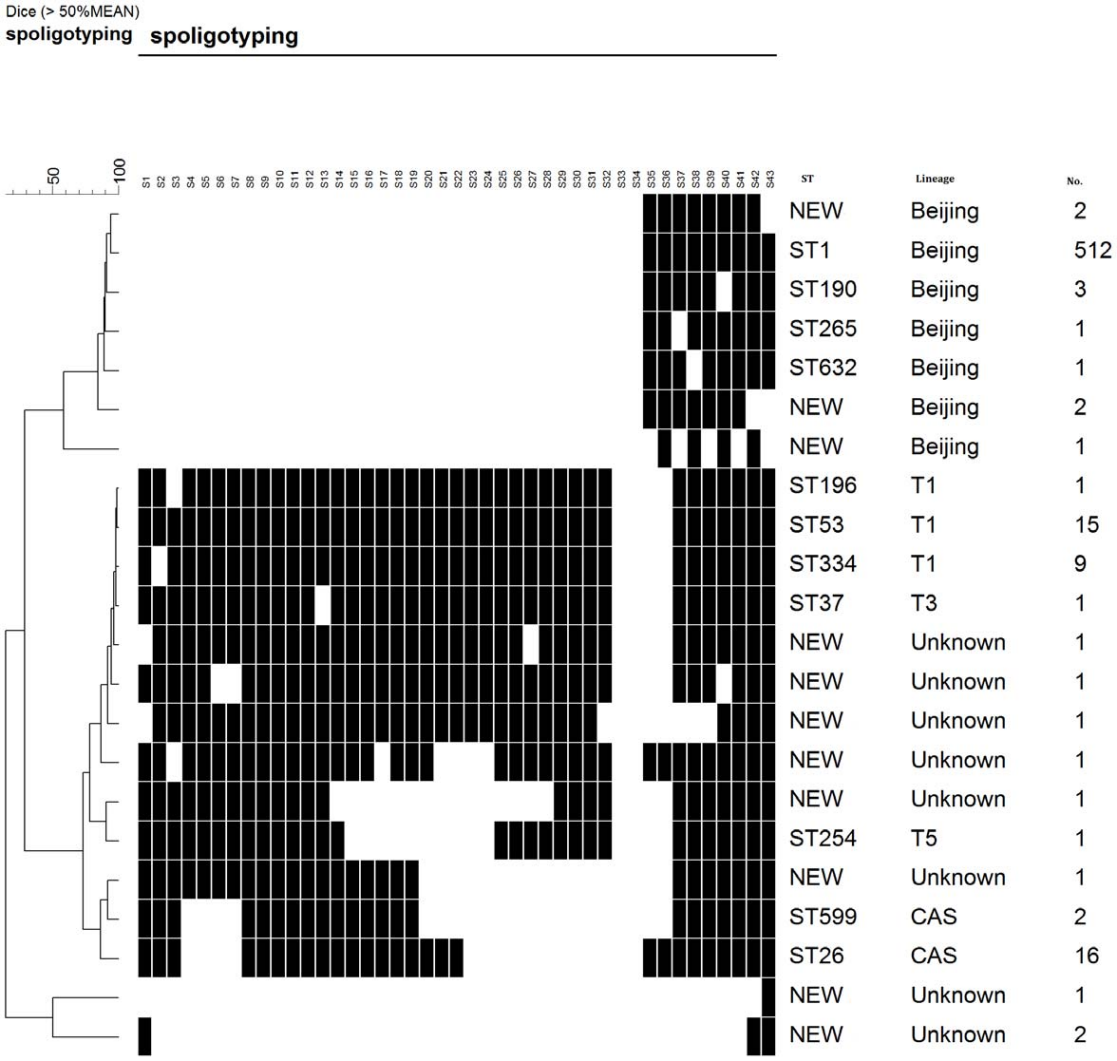
Spoligotypes of the 576 *M. tuberculosis* isolates. From left to right: 1) UPGMA dendrogram generated by 22 spoligotypes. 2) spoligotying patterns. 3) Spoligo-International-Type number. 4) Genetic lineage according to SITVIT2 database. 5) Strain numbers.

In terms of spoligotyping, we recognized a total of 22 distinct spoligotypes among the 576 isolates (Figure 2). Comparison of the spoligotyping results with the SITVIT2 database and application of the published rules for definition of the Beijing lineage (hybridized to at least three of the spacers 35 to 43 in the genomic direct-repeat region and showed an absence of hybridization to spacers 1 to 34) [7], permitted us to assign 567 isolates to 3 known spoligotype lineages, whereas 9 isolates could not be matched to any, and are, thus referred to as ‘new’ (Figure 2).

Clustering analysis revealed that 563 isolates were grouped into 9 clusters containing 2 to 512 isolates, while the other 13 isolates inhibited unique spoligotypes. The largest spoligotype lineage was Beijing genotype(90.63%, 522 isolates), most (512 strains) of which belonged to the classical Beijing genotype with a pattern that depicted the absence of the first 34 spacer oligonucleotides and the presence of spacers 35 to 43 [7], [15]. The next most common lineage was the ill-defined T lineage with 27 strains (4.69%), followed by CAS lineage with 18 strains (3.13%). The high rate of clustering (96.18%) was due to the lower discriminatory power of spoligotyping for the Beijing genotype.

Furthermore, there were no statistical significant associations between the Beijing lineage and age (*P*>.05), sex (*P*>.05), and treatment history (*P*>.05) (Table 1) in our study.

**Table 1:** Statistic analysis between Beijing genotype and sex, age, and treatment status.

	No.	No. of Beijing genotype	OR	P	95%CI
**All patients**	576	522			
**Sex**					
Male	329	298	1		
Female	247	224	0.983	1.000	0.558–1.731
**Age**					
0–19	57	49	1		
20–39	340	312	0.561	0.211	0.242–1.300
40–59	143	129	0.678	0.457	0.268–1.716
60–	36	32	0.781	0.763	0.217–2.809
**TB treatment history**					
New patient	317	287	1		
Treatment previously	259	235	0.980	1.000	0.558–1.723

### 24-locus MIRU-VNTR

When using the 24-locus MIRU-VNTR method to typing the 576 isolates, the full set of results was obtained for 517 isolates. For 59 isolates, no PCR products were obtained at one or more loci. These observations remained consistent even after repeated testing. These findings might result from the chromosomal deletion, nucleotide polymorphism in the sequences complementary to PCR primers [16], or decreased quality of DNA. In this study, these cases whereby no PCR products were obtained were excluded from the cluster analysis.

In total, 24-locus MIRU-VNTR method differentiated 247 genotypes among the 517 isolates (Table S1). A total of 229 isolates had unique patterns and the remaining 228 formed 62 clusters (2 to 37 isolates per cluster). The allelic diversity of each MIRU-VNTR locus for 517 isolates was estimated by using the Hunter-Gason discriminatory index (HGDI) (Table 2). The discriminatory power for 2 loci (QUB11b and MIRU31) exceeded 0.6 and these were regarded as highly discriminatory [17]. Five loci (QUB26, Mtub21, QUB4156, MIRU26 and MIRU20) showed moderately discrimination (0.3≤h≤0.6). Other loci were found to be less polymorphic, with HGDI within the range of 0 to 0.3. Locus MIRU24 was monomorphic.

**Table 2:** HGDI of the 24 MIRU-VNTR loci for the whole sample and for the Beijing genotype isolates.

	ETRA	ETRB	ETRC	MIRU2	MIRU4	MIRU10	MIRU16	MIRU20	MIRU23	MIRU24	MIRU26	MIRU27
Whole sample	0.169	0.109	0.110	0.003	0.097	0.193	0.222	0.413	0.031	0.000	0.482	0.078
Beijing genotype	0.089	0.030	0.049	0.000	0.082	0.030	0.171	0.434	0.034	0.000	0.430	0.056

	MIRU31	MIRU39	MIRU40	Mtub04	Mtub21	Mtub29	Mtub30	Mtub34	Mtub39	QUB11b	QUB26	QUB4156c
Whole sample	0.663	0.243	0.251	0.278	0.539	0.014	0.151	0.034	0.257	0.75	0.583	0.560
Beijing genotype	0.611	0.141	0.211	0.224	0.488	0.015	0.034	0.030	0.165	0.714	0.558	0.554

### Determination of a minimal set of MIRU-VNTR loci for differentiating Beijing genotype strains

To identify a minimal set of MIRU-VNTR loci for differentiating Beijing genotype strains in Tibet, the allelic diversity of each MIRU-VNTR locus were calculated separately (Table 2). When comparing the allelic diversity among all the isolates and Beijing genotype strains, 21 of the 24 MIRU-VNTR loci showed lower allelic diversity among Beijing genotype strains and were consistent with the close genetic relationships of those strains. The 3 loci that showed more allelic diversity among Beijing genotype strains were MIRU20, MIRU23 and Mtub29. Two loci (MIRU2 and MIRU24) were conserved among all Beijing genotype strains (h = 0.000).

Based on the allelic diversity of each MIRU-VNTR locus among Beijing genotype strains, the cumulative HGDI of the MIRU-VNTR locus combination was calculated and compared (Table 3). The cumulative HGDI of 22-locus VNTR was equal to that of the 24-locus MIRU-VNTR. The top 12 MIRU-VNTR loci combination appears to provide an HGDI close to that of the 24-locus MIRU-VNTR method.

**Table 3:** The cumulative HGDI with successive addition of each MIRU-VNTR locus.

Locus combination	VNTR alias	VNTR locus	No. of patterns	No. of clusters	No. of clustered isolates	No. of isolates in each cluster	Clustering rate (%)	HGDI (cumulative)
1	VNTR2163	QUB11b						
2	VNTR3192	MIRU31	24	17	466	2–135	94.9	0.8590
3	VNTR4052	QUB26	69	35	439	2–71	85.4	0.9332
4	VNTR4156	QUB4156	96	44	421	2–65	79.7	0.9506
5	VNTR1955	Mtub21	119	50	404	2–64	74.8	0.9582
6	VNTR2059	MIRU20	125	50	398	2–63	73.57	0.9598
7	VNTR2996	MIRU26	158	57	372	2–52	66.59	0.9713
8	VNTR0424	Mtub04	175	62	360	2–48	63.00	0.9751
9	VNTR0802	MIRU40	189	60	344	2–48	60.04	0.9784
10	VNTR3690	Mtub39	196	60	337	2–44	58.56	0.9805
11	VNTR1644	MIRU16	207	62	328	2–41	56.24	0.9830
12	VNTR4348	MIRU39	222	61	312	2–39	53.06	0.9850
13	VNTR2165	ETR A	228	63	308	2–39	51.80	0.9855
14	VNTR0580	MIRU4	233	63	303	2–38	50.74	0.9860
15	VNTR3007	MIRU27	235	64	302	2–38	50.32	0.9863
16	VNTR0577	ETR C	239	62	296	2–37	49.47	0.9867
17	VNTR2401	Mtub30	241	61	293	2–37	49.05	0.9869
18	VNTR2531	MIRU23	242	61	292	2–37	48.84	0.9870
19	VNTR3171	Mtub34	244	61	290	2–37	48.41	0.9872
20	VNTR2461	ETR-B	245	62	290	2–37	48.20	0.9875
21	VNTR0960	MIRU10	246	62	289	2–37	47.99	0.9876
22	VNTR2347	Mtub29	247	62	288	2–37	47.78	0.9877
23	VNTR0154	MIRU2	247	62	288	2–37	47.78	0.9877
24	VNTR2387	MIRU24	247	62	288	2–37	47.78	0.9877

## Discussion

Our results demonstrated that the population structure of *M. tuberculosis* isolates in Tibet appears to be very homogeneous, as only 3 spoligotype lineages were obtained for the 576 isolates, with 90.63% of the isolates belonging to the Beijing genotype. The predominance of the Beijing genotype in China is well documented in other studies. It is prevalent in Beijing (80 to 92.6%), Tianjin (91.7%), Heilongjiang (89.5%), Jilin (89.9%), and Shanghai (89%), but less prevalent in Guangxi (55.3%), Fujian (54.5%), and Guangdong (25%) [15], [18], [19], [20], [21]. Hence, Tibet is one of the regions where the proportion of the Beijing genotype is the highest. Owing to the special geographic and living habit, the predominance of a narrow range of genotypes might imply their long-standing presence in Tibet and maybe linked with limited contact with other populations.

Our results showed that there was no association between the prevalence of Beijing genotype and sex, age, and treatment status. This indicated that maybe there were other factors that contributed to the spread of Beijing genotype strains. Demographic factors may be responsible for the dominance of Beijing genotype strains based on a co-evolution between the host and the pathogen [22], [23]. This estimation for the correlation between the Beijing genotype and sex, age, and treatment status were, perhaps, biased by a smaller sample size of non-Beijing genotype strains compared to Beijing genotype strains.

We found that the spoligotyping method could not effective distinguish Beijing genotype strains. Therefore in our study, these strains were further subjected to the newly proposed 24-locus MIRU-VNTR method and we found that the allelic diversity of the VNTR loci varied significantly at each locus. Among the 24 loci we investigated, QUB11b and MIRU31 were highly discriminative (h≥0.6), QUB4156, Mtub21, MIRU20 and MIRU26 were moderately discriminative, and other loci were poorly discriminative. When scrutinizing the 24-locus scheme, we found that MIRU24 and MIRU2 remained monomorphic in every published setting in China, Japan and Russia [20], [24], [25]. Locus MIRU24 remained monomorphic, which mirrorsi the previous observation that this locus is phylogenetically conserved.

When comparing the HGDI of this locus set with the VNTR loci reported in other areas (Table 4) [20], [24], [25], [26], [27], [28], [29], [30], we found that the allelic diversity of these loci were different from that described in other reports related to analyses of Beijing genotype strains. Many lowly polymorphic loci (MIRU31 and MIRU20) from Beijing, Shanghai, Wuhan, Hong Kong, and Russia may be able to discriminate Beijing genotype strains in Tibet. Most of the VNTR loci showed higher discriminatory power for Japan than China and Russia. A generally lower discriminatory power of VNTR loci for Russia may be considered a reflection of the recent clonal expansion of the Beijing genotype strains in that country. Although MIRU-VNTR loci showed a variation in the ability to differentiate Beijing genotype strains from different geographical areas, this may be attributed to the dissimilarities in the population structure of the circulating *M. tuberculosis* strains in distinct geographic areas.

**Table 4:** Allelic diversity of different MIRU-VNTR in Beijing genotype strains from different locations.

VNTR alias	Tibet, China(this study)	Beijing, China	Heilongjiang China	Shanghai, China	Wuhan, China	Hong Kong,China	Hong Kong, China	Kobe, Japan	St. Petersburg, Russia
QUB11b	0.694	0.651	0.704	0.655		0.669	0.618	0.772	0.205
MIRU31	0.617	0.169	0.395	0.246	0.23	0.156	0.200	0.322	0.160
QUB26	0.525	0.518	0.607	0.595		0.314	0.299	0.741	0.636
QUB4156	0.519	0.395	0.182	0.492		0.167		0.611	0.082
Mtub21	0.491	0.556	0.396	0.523				0.393	0.330
MIRU20	0.440	0.014		0.061	0.25			0.022	0.120
MIRU26	0.429	0.353	0.596	0.612	0.60		0.200	0.383	0.520
Mtub04	0.224	0.306	0.391	0.297				0.459	0
MIRU40	0.221	0.194	0.292	0.147	0.23		0.196	0.327	0.122
Mtub39	0.166	0.171	0.174	0.061				0.186	0
MIRU16	0.158	0.068	0.200	0.242	0.55		0.058	0.310	0.082
MIRU39	0.147	0.119	0.290	0.286	0.03		0.320	0.221	0
ETR A	0.090	0.232	0.238	0.031		0.188	0.201	0.147	0.158
MIRU4	0.066	0.120	0.212	0.061	0.08	0.072	0.019	0.086	0
MIRU27	0.058	0.014		0.031	0.08			0.115	0
ETR C	0.054	0.094				0.057	0.165	0.022	0.042
Mtub30	0.033	0.068	0.133	0.091				0.403	0.042
MIRU23	0.033	0.014		0.061	0.25			0.176	0
Mtub34	0.029	0.014		0.089				0.065	0
ETR B	0.029	0.014		0		0.064	0	0	0
MIRU10	0.025	0.144	0.154	0.195	0.52		0.377	0.419	0.082
Mtub29	0.013	0.119	0.123	0.061				0.043	0.087
MIRU2	0	0		0	0			0	0
MIRU24	0	0		0	0			0	0

The 24-locus MIRU-VNTR scheme has some technical limitations: in China, MIRU-VNTR can be only performed manually and, consequently, was very time-consuming and tedious for this study. Some reports demonstrated that combination of 24-locus MIRU-VNTR and spoligotyping could improve the discriminatory power of *M. tuberculosis* strains. In addition, not all 24 loci are required for genotyping *M. tuberculosis* strains in any given situation because the number of loci required depends on the population structure of *M. tuberculosis*. In this study, the top 12 loci also demonstrated a high discriminatory power among Beijing genotype strains. A 12-locus VNTR typing method proposed in Japan is reported to be appropriate for discrimination of Beijing strains [31], but the discriminatory power of these loci sets require further study in different settings for other areas.

In conclusion, Beijing genotype strains appeared to be widely disseminated across Tibet. The analysis of MIRU-VNTR data might be useful to select appropriate VNTR loci for the genotyping of *M. tuberculosis*.

## Materials and Methods

### Ethics statement

The study was approved by the Ethics Committee of National Institute for Communicable Disease Control and Prevention, Chinese Center for Disease Control and Prevention. All patients in the study signed informed consent form.Clinical isolates and DNA samples.

We randomly collected a total of 590 *M. tuberculosis* isolates between 2006 and 2010 from 590 Tibetan patients at seven different regional Center of Disease Prevent and Control (CDC) in Tibet (Lhasa, Xigaze, Nyingchi, Sannan, Nagqu, Changdu, and Ngari). Chromosomal DNA was extracted by boiling a loopful of colonies from L-J slants in 400 µL of 10 mM tris-HCl and 1 mM EDTA (pH8.0) buffer for 10 minutes. The suspension was centrifuged at 12000 rpm for 10 minutes, and the supernatant was stored at −20°C until further use [32]. Fourteen isolates were excluded because of sample contamination and/or mixed infection as detected by double alleles in two or more MIRU-VNTR loci [10], [33]. A final sample of 576 isolates was retained for analysis.

### Molecular typing methods

We performed spoligotyping according to a standard protocol as described by Kamerbeek et al [4]. We entered the results in an Excel spreadsheet in a 43-digit binary format representing the 43 spacers [5] and compared them to SITVIT2 database (an international spoligotype database at the Institute Pasteur de Guadeloupe), which is an updated version of the published SpolDB4 database [5].

In terms of the 24-locus MIRU-VNTR typing, we used PCR primers flanking each of the 24 loci that were described by Supply et al [10]. Each MIRU-VNTR locus was amplified individually in a 25-µL reaction volume in a 0.2-ml PCR tube. PCR products were analyzed by electrophoresis on a 2% agarose gel using 100 bp DNA ladder as size markers. The H37Rv strain was run as an additional control for accuracy. Sizing of the PCR fragments and assignment of the VNTR alleles were done using Bionumerics software version 5.0 (Applied Maths, Sint-Martens Laten, Belgium). Clusters were defined when 100% similarity was observed between patterns.

To minimize the risk of laboratory cross-contamination, DNA extraction and PCR amplification were conducted in separate rooms. The PCR laboratory has four disconnected rooms for preparation of the PCR mixtures, addition of the DNA, PCR amplification, and electrophoretic fractionation. Negative controls (sterile water) were included to control for cross contamination.

### Analysis of genotyping

Bionumerics software version 5.0 and MIRU-VNTRplus (http://www.miru-vntrplus.org) were used to analyze genotyping data [34]. Clustering analysis was done using the unweighted pair group method with arithmetic averages (UPGMA). The Dice and categorical coefficients were used in spoligotyping and MIRU-VNTR, respectively. The Hunter-Gaston discriminatory index (HGDI) was used to evaluate the discriminatory power of the typing methods and the allelic diversity of the VNTR loci [35]. The clustering rate was defined as (n_c_ - c)/n, whereby n_c_ is the total number of clustered cases, c is the number of clusters, and n is the total number of cases in the sample [36].

The chi square test was used to assess association of Beijing genotye with sex, age, and treatment status by using SPSS 11.5 (SPSS Inc., Chicago, IL, USA). *P*<.05 was defined as statistically significant.

## Supporting Information

Table S1 **24-locus MIRU-VNTR profile and spoligotyping profile of 517 isolates.** The table provides the 24-locus MIRU-VNTR typing data and spoligotpying data in 517 isolates.
(XLSX)
